# 1005. Case Study. Reconstruction of a Fourth Degree Scalp Burn Using Biodegradable Temporizing Matrix: A Case Report

**DOI:** 10.1093/jbcr/irag033.193

**Published:** 2026-04-06

**Authors:** Megan E Molnar, Kyra Leonard

**Affiliations:** Westchester Medical Center, Tarrytown, NY; Westchester Medical Center, NY, New York

## Abstract

**Patient Presentation (age range, injury details, relevant history):**

78 year-old female with past medical history of hypertension, hyperlipidemia and hypothyroidism who sustained 33.5% TBSA mixed third and fourth degree contact burns to the head, neck, right upper extremity, left flank, and bilateral lower extremities in a motor vehicle accident. The patient accidentally collided with a trunk that was carrying asphalt, and the asphalt leaked into the patient's car. Extrication time was 15 minutes. The patient was taken to the emergency department for evaluation and activated as a level 2 trauma. The patient underwent CT scans of the head, cervical spine, thoracic and lumbar spines, and thorax, abdomen and pelvis as per trauma team recommendations, and was found to have compression fractures of the L1 and L4 vertebrae which were stable on upright xray and required no acute intervention. The patient was admitted to the burn surgery service for burn resuscitation and wound care.

**Clinical Challenges:**

Among her other injuries, the patient sustained a fourth degree burn to the right parieto-occipital scalp, requiring surgical debridement of the skull. Thus, the patient required optimization of her scalp wound bed to allow for successful coverage via autografting.

**Management Approach:**

On admission, the patient's scalp wounds were dressed with collagenase and gentamicin, wrapped with sterile bandages, and changed daily until excision. On hospital day 6, the patient underwent tangential excision of the burn wounds on her right parieto-occipital scalp with biodegradable temporizing matrix (BTM) placement. The patient underwent local wound care with collagenase and gentamicin to her scalp wound until hospital day 23, when she was taken to the OR for re-evaluation of her scalp wound. Intraoperatively, the exposed right parieto-occipital skull was still poorly perfused, consistent with fourth degree burn injury. The wound was debrided with bone saw down to punctate bleeding. Hemostasis was achieved and BTM was applied directly to the skull. On hospital day 42, the BTM was removed in the OR, and the occipital wound had granulated well, with no visible bone. Granulation tissue was gently debrided and 1:1 split thickness skin graft was placed. Scalp wound was dressed with gentamicin and bulky dressing, which was left intact until postoperative day 5.

**Outcomes:**

The patient's right parieto-occipital scalp autograft had 100% take on postoperative takedown. No graft loss was noted on daily bedside dressing changes throughout the rest of her admission.

**Lessons Learned:**

This case study demonstrates that BTM is an effective means of promoting granulation tissue formation to achieve a suitable wound bed for autograft placement. Patients with similar full thickness or fourth degree burns to the head and skull in our institution have required plastic and reconstructive surgical consultation for coverage, including omental harvest with omental flap creation.

**Applicability to Practice:**

Use of BTM in fourth degree burn injury reconstruction to the head and skull may reduce the need for invasive or higher risk tissue coverage procedures and increase graft take.

